# Bioassays of *Beauveria bassiana* Isolates against the Fall Armyworm, *Spodoptera frugiperda*

**DOI:** 10.3390/jof8070717

**Published:** 2022-07-08

**Authors:** Atif Idrees, Ayesha Afzal, Ziyad Abdul Qadir, Jun Li

**Affiliations:** 1 Guangdong Key Laboratory of Animal Conservation and Resource Utilization, Guangdong Public Laboratory of Wild Animal Conservation and Utilization, Institute of Zoology, Guangdong Academy of Sciences, Guangzhou 510260, China; atif_entomologist@giabr.gd.cn (A.I.); ayeshaafzal403@yahoo.com (A.A.); 2 Institute of Molecular Biology and Biotechnology, The University of Lahore, 1-Km Defense Road, Lahore 54000, Pakistan; 3 Honeybee Research Institute, National Agricultural Research Centre, Park Road, Islamabad 45500, Pakistan; zaqadir@parc.gov.pk; 4 Department of Entomology and Wildlife Ecology, University of Delaware, Newark, DE 19716, USA

**Keywords:** *Beauveria bassiana*, eggs, neonate, biological control, fall armyworm

## Abstract

The control of *Spodoptera frugiperda*, the key invasive pest of maize, is a serious concern due to its biology and the current global restriction on applying synthetic pesticides. Entomopathogenic fungi are considered to be a potential biological control strategy. The pathogenicity of 12 isolates of *Beauveria bassiana* in the immature stages and feeding efficacy of *S. frugiperda* were evaluated. The *B. bassiana* isolates QB-3.45, QB-3.46 and QB-3.428 caused the highest egg mortality rates of 87.3, 82.7 and 79.3%, respectively, when applied at a concentration of 1 × 10^8^ conidia/mL and measured at 7 days post-treatment. Neonate mortality rates of 45.6 to 53.6% were observed with the same isolates. The *B. bassiana* isolates caused significant cumulative mortality rates ranging from 71.3 to 93.3% at 14 days post-treatment and reduced larval feeding efficacy from 69.4 to 77.8% at 48 h post-treatment. This study supports using the effective *B. bassiana* isolates as a biological control agent against *S. frugiperda.* The significant mortality of the eggs and neonatal larvae and the reduction in the feeding efficacy of the second instar larvae of the *S. frugiperda* that were treated with isolates of *B. bassiana* supports the application of entomopathogenic fungi as a biocontrol agent for the effective control of the *S. frugiperda* population.

## 1. Introduction

Maize is one of the primary crops producing commercial cereal commodities worldwide, second to rice and wheat [1]. In addition to feeding animals and humans, it is also used in the paper, bioplastic, beverage, and textile industries [2]. Maize is one of the most economical crops in China [3], which was ranked as the second-greatest country in the world for producing and consuming maize, after the United States [4]. One-fifth of the yield of the country’s total maize is produced in the northeast region [5].

Fall armyworm (FAW), *Spodoptera frugiperda*, JE Smith, 1797, (Lepidoptera: Noctuid) is one of the most destructive, invasive pests with more than 350 host-plant species due to its polyphagous nature; however, it prefers to feed on maize. FAW is native to the Americas [6,7]. Maize crops have been severely threatened due to the recent invasion of invasive FAW in many countries, especially in China [8,9], which has raised the serious issue of national food security [10]. FAW larvae feed on the young leaves, leaf whorl, or tassels and might cause severe yield loss if they are not controlled in a timely fashion [11,12].

FAW is considered an A1 quarantine invasive pest in Africa [13]. In 2019, annual yield losses of up to 6.2 billion tons were caused by FAW damage to maize in more than 44 African countries [14]. FAW has caused 16 to 52% damage to fodder maize in India [15], 67 and 22% damage to maize in Zambia and Ghana, respectively [16], and 47 and 32% yield losses of maize in Kenya and Ethiopia, respectively [17]. This has ultimately led to 4.66 billion dollars in economic losses for Africa [18].

The fall armyworm was first detected in Africa and it then expanded throughout the world to locations such as Nepal, Indonesia, and Swaziland [19,20,21]. FAW was firstly reported in China on 11 December 2018 [22,23,24], it later invaded 26 provinces of the southern region [24,25] and caused serious damage to multiple crops [26]. The seedling and flowering stages of the peanut were damaged by 78 and 65%, respectively [27] and barley, wheat, tobacco, and fields have also been severely affected by FAW [9,28,29].

Most maize growers in Indonesia prefer using pesticides to control this invasive pest in order to avoid the yield losses of maize to FAW [30]. The continuous application of chemical pesticides leads to the development of pesticide resistance in insect pests all over the world, especially in China [31,32]. Pesticide-based management practices are not only considered to be unsustainable but it is also known that they negatively impact the ecosystem, humans and the natural predators of pests [33,34]. Therefore, many studies have focused on developing environmentally safe strategies for the management of arthropod pests [35,36]. Currently, biopesticides are one of the most sustainable and economical practices that could be a key component of integrated pest management [37,38,39,40,41]. A strong association has been reported between many pathogens, including viruses and fungi, with FAW [42], but only a few among them have been able to infect to the pest directly or indirectly [43,44,45,46,47,48,49].

Biocontrol agents, such as entomopathogenic fungi, have prominent virulence factors and are rapidly emerging as prime substitutes for synthetic insecticides [50]. *Metarhizium anisopliae*, *Beauveria bassiana* and *Bacillus thuringiensis* are the most effective biocontrol agents among the active biopesticide products that have been registered by the United States Environmental Protection Agency as of 2016 [51]. The most commonly used biopesticide is *B. bassiana*, which can be effectively transmitted and produces a variety of toxin-causing infections in the host [52,53]. *B. bassiana* was first isolated from silkworm cadavers by Agostino Bassi in the 19th century and it has since infected more than 200 species of insects belong to different orders [54].

*Beauveria bassiana* is competitive with synthetic insecticides against forest and farm insect pests in China [55]. Six isolates of *B. bassiana* were screened in order to evaluate their pathogenicity against the *S. frugiperda* adult in Nairobi, Kenya [56]. Tomato plants that were treated with the *B. bassiana* resulted in affecting the larval development of *S. frugiperda* in Eldoret, Kenya [57]. Entomopathogenic fungi can strengthen Integrated Pest Management (IPM) programs and reduce the environmental impact of pesticides against *S. frugiperda* in Mexico [58,59]. The isolates of *B. bassiana* proved to be effective for the management of *S. frugiperda* in Thailand [60]. *B. bassiana* isolates are considered to be a sound biocontrol agent for the management of FAW [61,62].

Among the identified species of fungi, the most utilized are in the genus *Beauveria*, from which several bio-insecticides have been derived and used in Indonesia [63]. Entomopathogenic fungi (EPF) will ensure a healthy environment and a food-secure world when they are used as a biological method of pest control [64]. Twelve *B. bassiana* isolates were used in the present study, originating from different hosts and already tested for their virulence against *Solenopsis invicta* Buren, one of the most important pests in China [65]. *B. bassiana* was indicated to be an entomopathogen in many insects’ orders; lepidopteran pests in particular have been effectively controlled by this fungus [66]. Three different doses were tested in the present study as is the ideal method to use when testing any fungal isolate against any agricultural insect pest. Indeed, many previous studies have already used multiple doses to assess the virulence of EPF [67]. Consequently, there is a dire need to develop an alternative and sustainable strategy to suppress FAW.

Entomopathogenic fungi (EPF) are environmentally friendly and could replace synthetic insecticides. However, one negative impact of the EPFs is that they kill or infect the insect much more slowly than a synthetic insecticide due to their different modes of action depending on which insect continuously feeds on the crop. The different life stages of any insect do not respond to any pathogenic stress in the same way. So, it is very normal to test EPF on various life stages in order to understand which life stage is most susceptible or to find the stage that is the most resistant to the said fungal isolate. This method is able to inform the users of the most appropriate stage of the insect to target for effective biological control programs. Our previous study has concluded that the eggs are the most susceptible stage for targeting as compared to the other life stages of FAW [61,62]. It has further been confirmed that EPF have the potential to infect any stage of the insect’s life; however, this does not mean that all of the insect’s stages are equally susceptible to pathogenic infection [68].

These isolates of *B. bassiana* were proved to be pathogenic against *S. invicta* in 2018 [65] but afterwards no further research work has been done on the use of these isolates against other insect pests. Little information is available about the virulence of these isolates of *B. bassiana* against the immature stages (eggs, neonates, larvae and pupae) and the feeding efficacy of FAW. Therefore, the present study aims to investigate the bioassay of isolates of *B. bassiana* for the management of FAW.

## 2. Materials and Methods

### 2.1. Insect Rearing

The FAW eggs were established using a collection of larvae that were found on maize crops from Guangdong province, China, during the maize growing season. The collected larvae were cultured on an artificial diet [69] and the resultant adults were fed artificial diets of a 10% honey solution soaked into sterile cotton balls. The larvae were kept under laboratory conditions at 25 ± 2 °C, with a photoperiod of 12:12 (dark: light) and 65% ± 5% relative humidity (RH). Once oviposited, the egg batches were maintained in an environmental growth chamber under laboratory conditions and, upon hatching, the neonatal larvae were shifted to a transparent rectangular plastic box (28 × 17 × 18 cm). The FAW larvae were obtained from uniform culture following 35 generations of laboratory mass rearing [61] at the Institute of Zoology, Guangdong Academy of Sciences (IZ-GDAS).

### 2.2. Preparation of B. bassiana Isolates

The pathogenicity of 12 isolates of *B. bassiana* was evaluated against the immature stages and the feeding efficacy of FAW. *B. bassiana* isolates that were obtained from the IZ-GDAS’s laboratory were cultured on potato dextrose agar (PDA) and maintained at 25 ± 2 °C in the dark. The tested isolates of *B. bassiana* were previously identified by Li et al. [65] using their cultural and morphological characteristics. Macroscopic observations were assessed with colony characteristics, i.e., shape, growth pattern and color. Molecular analysis was carried out using multiplex PCR in order to verify the morphological identification of the tested isolates of *B. bassiana*. The information regarding the *B. bassiana* isolates is available in (Table 1).

The fungal conidia were suspended in a 10 mL distilled water volume with 0.05% Tween-80 in a universal bottle containing glass beads. The fungal conidia suspension was vortexed for five minutes at 700 rpm in order to break the conidial clumps so as to ensure homogenous suspension. Using a hemocytometer, the conidial suspensions were adjusted to concentrations of 1 × 10^6^, 10^7^ and 10^8^ conidia/mL [59]. A dose-dependent mortality effect has already been observed in previous laboratory studies by many authors, so as such three doses were selected in the present study in order to investigate if there is any different observation between this study with multiple doses and previous studies that used multiple doses to assess the virulence of *B. bassiana* isolates against FAW [60,61].

Prior to the start of the experiments, germination tests were also conducted [70]. The length of the germ tube was equal to twice the conidium diameter indicated at the germination of the conidia [71,72]. The twelve isolates of *B. bassiana* showed ≥90% germination rate 18 h post-incubation at 25 ± 2 °C (Supplementary Table S1).

### 2.3. Pathogenicity of B. bassiana Isolates against Eggs and Neonate Stage of FAW

FAW eggs (1 to 2 days of age) were obtained from the adult cylindrical cages. A batch of 50 eggs was separated with a light microscope using a camel hairbrush. Then, 10 mL of the spore suspension at each concentration was sprayed on a set of 50 eggs using manual atomized spray bottles (20 mL). A sterilized paper towel was used at the bottom of the Petri plate for the absorption of the extra spore suspension. The control was treated with distilled water containing 0.05% Tween-80. The batch of treated eggs was air-dried for 1 h under a laminar hood. The eggs (hatched and non-hatched) were recorded at 7 days post-treatment.

The FAW neonatal larvae that emerged from the eggs that were treated with the isolates of *B. bassiana* were fed fresh maize leaves in a rectangular plastic box (28 × 17 × 18 cm^3^) that was lined with moist filter paper, incubated at 25 ± 2 °C and monitored daily. The maize was grown under lab conditions in order to ensure that the maize leaves were fresh and insecticide-free.

The mortality of the neonatal larvae that were treated with the isolates was further recorded daily for 7 days post-emergence. The bioassays were laid out according to a completely randomized design with three replications for each treatment. Mycosis was assessed for the dead cadavers by following the approach of Aktuse et al. [60]. The dead larvae were surface-sterilized with 70% alcohol and rinsed thrice in distilled water. The surface-sterilized dead larvae were kept in Petri dishes containing sterile filter paper in order to observe their fungal growth. The mortality due to the treated fungal isolate was confirmed by the presence of hyphae and conidia on the bodies of dead larvae. The cumulative mortality of both the neonates and larvae of the FAW was calculated by counting the dead eggs and neonatal larvae divided by a total number of eggs at 14 days post-treatment [61].

### 2.4. Pathogenicity of B. bassiana Isolates against Second Instar Larvae of FAW

Fresh maize leaves were placed individually in a perforated rectangular plastic box (28 × 17 × 18 cm^3^). A group of 30 second instar larvae of FAW was shifted into the box containing the maize leaves. Each group of 30 larvae represented one replicate and three replications were used for each treatment with a completely randomized design. Each group of second instar larvae in each plastic box was provided with fresh maize leaves that had been treated with 10 mL of 1 × 10^6^, 10^7^ or 10^8^ conidia/mL using a spray tower. Each concentration was sprayed on every second instar larva to ensure that none escaped from the fungal spore suspension by hiding beneath the leaves’ surfaces. The control larvae were treated with distilled water (0.05% Tween-80). The treated larvae were incubated at 25 ± 2 °C and fresh maize leaves were provided daily. The larval mortality rate was recorded daily for 7 days. A mycosis test was conducted in order to confirm that the mortality of the larvae was due to a fungal infection, as described above.

### 2.5. Pathogenicity of B. bassiana Isolates against Feeding Efficacy of Second Instar Larvae of FAW

A group of 15 second instar stages of FAW was treated with 10 mL of 1 × 10^6^, 10^7^ or 10^8^ conidia/mL using a manual atomizer sprayer bottle (20 mL) [59]. Fresh maize leaves (12 g) were placed in a rectangular plastic box (28 × 17 × 18 cm^3^). The control was treated with distilled water containing 0.05% Tween-80. The maize leaves that were provided to the group of second instar larvae as an insect diet were weighed before and after being treated. The feeding efficacy percentage was measured by calculating the total weight of the fresh maize leaves, in grams, that were provided to the larvae divided by the total weight of the leaves that were not fed on by the larvae, multiplied by 100 [61]. The feeding efficacy data were collected 24 and 48 h post-treatment. The bioassays were laid out according to a completely randomized design with three replications for each treatment.

### 2.6. Pathogenicity of B. bassiana Isolates against Pupae of FAW

The isolates of *B. bassiana* were screened for their ability to cause the mortality of the pupae of FAW. The pupae of the FAW were treated with 10 mL of 1 × 10^6^, 1 × 10^7^, or 1 × 10^8^ conidia/mL using manual atomizer sprayer bottles (20 mL). The FAW pupae were placed in a perforated rectangular plastic box (28 × 17 × 18 cm^3^). Each group of 10 pupae represented one replicate and three replications were used for each treatment. The control was treated with a solution of distilled water containing 0.05% Tween-80. The pupal mortality rate was recorded for 15 days [32]. A pupa was considered dead if it didn’t show even slight movement upon being touched nor did it turn black nor did it emerge within 15 days after pupation. All of the treatments were administered using a completely randomized design.

### 2.7. Data Analysis

The mortality data for all of the stages were analyzed using Abbott’s formula [73] and the Shapiro–Wilk [74] test was used to analyze the normality of all of the stages before they were subjected to a one-way analysis of variance (ANOVA) using Tukey’s highly significant difference (HSD) post-hoc test at a 95% level of significance. Factorial ANOVA was used to analyze the interaction of the various factors, including the fungal conidial concentrations, treatments and immature stages, and this was followed by Tukey’s highly significant difference (HSD) post-hoc test. SPSS (version 22.0) and Statistix^®^ version 8.1 (Analytical Software, Tallahassee, FL, USA) were used to perform for the data analysis and to calculate the homogeneous letters, respectively.

## 3. Results

### 3.1. Effect of B. bassiana Isolates on Eggs of FAW

**Figure 1:**
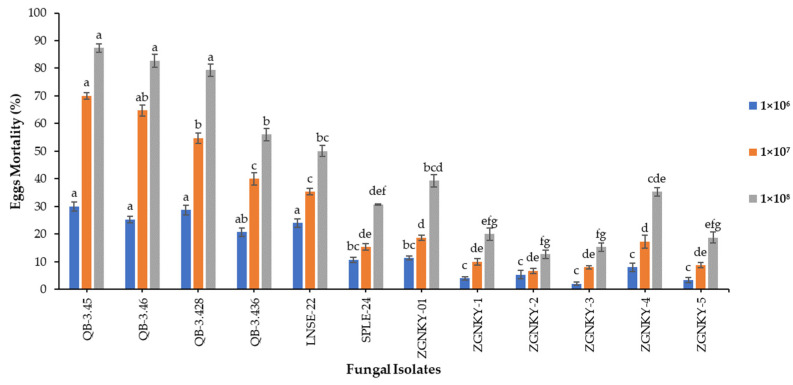
Mortality of FAW eggs infected with *B. bassiana* isolates. Means followed by the same letter of alphabets are not significantly different according to Tukey’s test at *p* < 0.05.

The results revealed that the QB-3.45 and QB-3.428 isolates of *B. bassiana* induced egg mortality rates of 30.0 and 28.7%, respectively, followed by the QB-3.46, LNSE-22 and QB-3.436 isolates, which caused 25.3, 24.0 and 20.7% FAW egg mortality when applied at a concentration of 1 × 10^6^ conidia/mL and measured at 7 days post-treatment (F_19.5_ = 12; *p* < 0.000). (Figure 1 and Supplementary Table S2). QB-3.45 and QB-3.46 were outperformed by causing egg mortalities of 70.0 and 64.7%, respectively, followed by the QB-3.428 isolate (54.7%) when the treatment concentration was 1 × 10^7^ conidia/mL (F_69.9_ = 12; *p* < 0.000). However, the QB-3.436 and LNSE-22 isolates caused egg mortality rates of 40.0 and 35.3% (Figure 1 and Supplementary Table S3). The QB-3.45, QB-3.46 and QB-3.428 isolates of *B. bassiana* induced the highest egg mortalities of 87.3, 82.7 and 79.3%, respectively, followed by the QB-3.436 and LNSE-22 isolates, which induced egg mortalities of 56.0 and 50.0%, respectively, when applied at a concentration of 1 × 10^8^ conidia/mL (F_60.3_ = 12; *p* < 0.000). However, the XJWLMQ-32 and SPLE-24 isolates of *B. bassiana* caused 39.3 and 30.7% egg mortality, respectively, 7 days post-treatment (Figure 1 and Supplementary Table S4).

### 3.2. Effect of Isolates against Neonate Larvae of FAW

**Figure 2:**
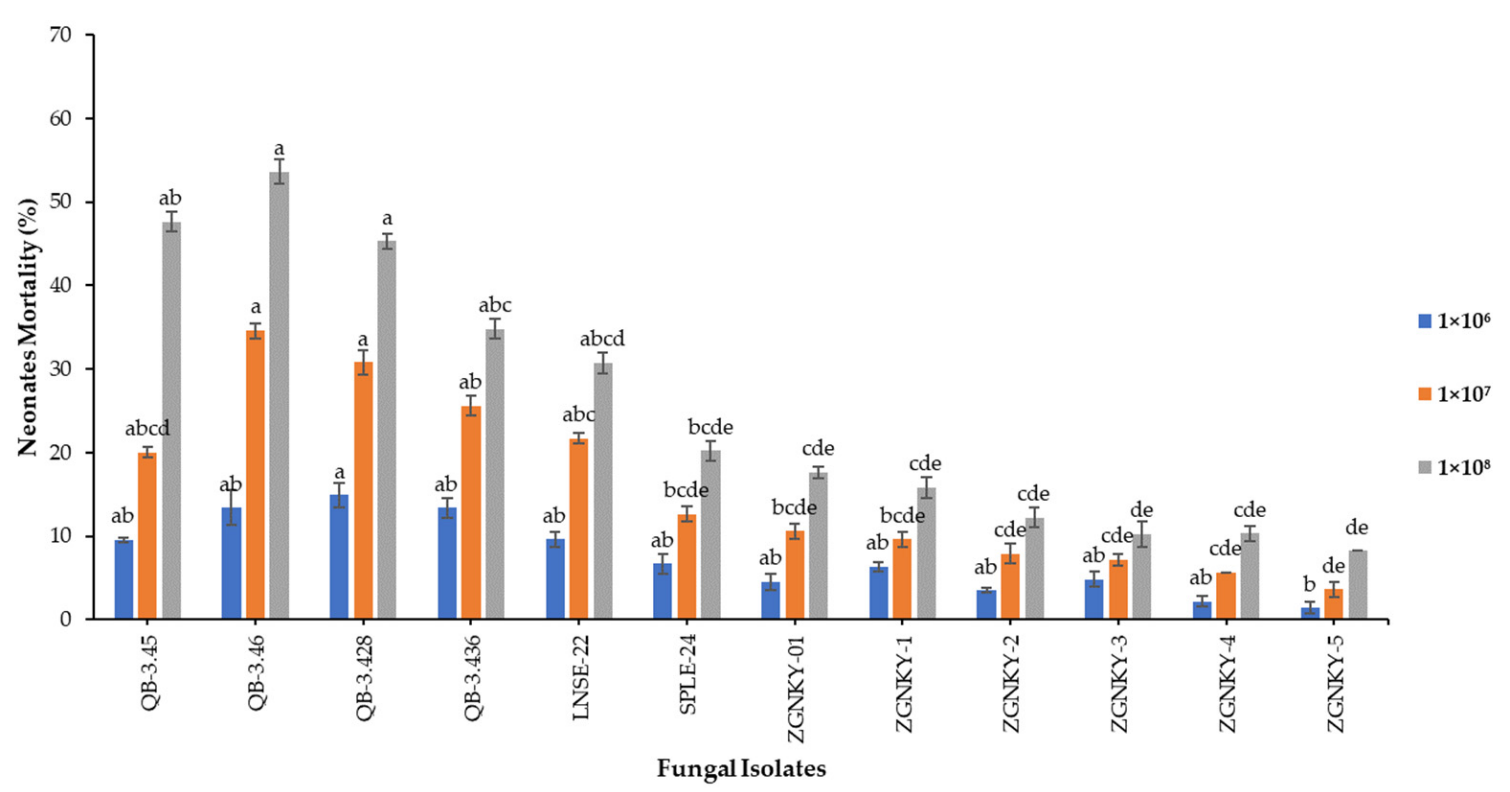
Mortality of neonatal larvae of FAW infected with *B. bassiana* isolates at the egg stage. Means followed by the same letters of alphabets are not significantly different according to Tukey’s test at *p* < 0.05.

The results indicated that the effects of the QB-3.428 and QB-3.46 isolates of *B. bassiana* were the least significant and induced additional mortality rates of 14.9 and 13.4% when applied at a concentration of 1 × 10^6^ conidia/mL (F_3.63_ = 20; *p* < 0.002) against the neonatal larvae of FAW and measured at 7 days post-treatment (Figure 2 and Supplementary Table S2). The QB-3.46 and QB-3.428 isolates of *B. bassiana* caused neonatal mortality rates of 34.6 and 30.8%, respectively, followed by QB-3.436, LNSE-22 and QB-3.45, which caused 25.6, 21.7 and 20.0% neonatal mortality, respectively, when applied at a concentration of 1 × 10^7^ conidia/mL (F_10.3_ = 12; *p* < 0.000) (Figure 2 and Supplementary Table S3). The QB-3.46, QB-3.45 and QB-3.428 isolates of *B. bassiana* induced neonatal mortality rates of 53.6, 47.6 and 45.3%, respectively, followed by QB-3.436 and LNSE-22, which caused 34.8 and 30.7% neonatal mortality rates, respectively, when applied at a concentration of 1 × 10^8^ conidia/mL (F_11.6_ = 12; *p* < 0.000) (Figure 2 and Supplementary Table S4). Furthermore, only 10% of the insect cadavers showed mycosis.

### 3.3. Effect of Beauveria bassiana Isolates on Cumulative Mortality

The results showed that the QB-3.428, QB-3.45 and QB-3.46 isolates of *B. bassiana* induced cumulative mortality rates of 39.3, 36.7 and 35.3% against eggs and neonates, respectively, followed by the QB-3.436 and LNSE-22 isolates’ mortality rate of 31.3% at a concentration of 1 × 10^6^ conidia/mL and measured at 14 days post-treatment (F_16.1_ = 20; *p* < 0.000). However, ≤16.7% cumulative mortality was observed for the other tested isolates (Supplementary Table S2). The *B. bassiana* isolates QB-3.46, QB-3.45 and QB-3.428 induced 77.3, 76.0 and 68.7% cumulative mortality rates, respectively, followed by the QB-3.436 and LNSE-22 isolates that caused cumulative mortality rates of 55.3 and 49.3%, respectively, when applied at a concentration of 1 × 10^7^ conidia/mL (F_52.5_ = 12; *p* < 0.000). Furthermore, the rest of the other isolates of *B. bassiana* caused ≤26.0% cumulative mortality rates (Supplementary Table S3). However, the QB-3.45, QB-3.46 and QB-3.428 isolates of *B. bassiana* induced the highest cumulative mortality rates of 93.3, 92.0 and 88.7%, respectively, followed by QB-3.436 and LNSE-22 which caused 71.3 and 65.3% cumulative mortality rates, respectively, when applied at a concentration of 1 × 10^8^ conidia/mL (F_90.3_ = 12; *p* < 0.000). Furthermore, 50.0 and 44.7% cumulative mortality rates were observed with the ZGNKY-01 and SPLE-24 isolates of *B. bassiana* (Supplementary Table S4).

### 3.4. Effect of Beauveria bassiana Isolates on Second Instar Larvae of FAW

**Figure 3:**
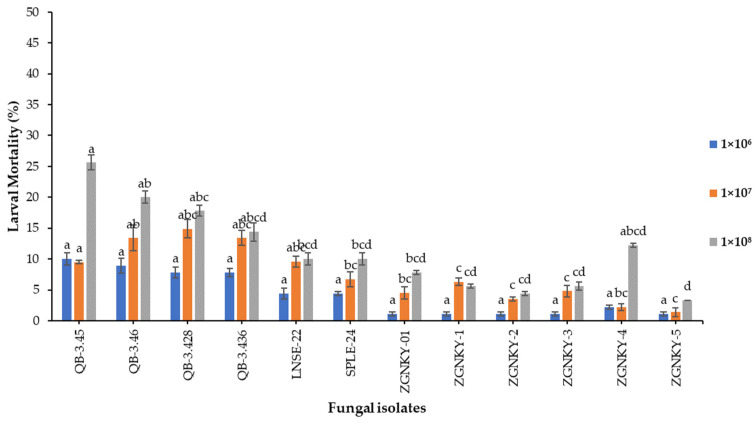
Mortality of second instar larvae of FAW infected with different concentrations of *B. bassiana* isolates. Means followed by the same letters of alphabets are not significantly different according to Tukey’s test at *p* < 0.05.

The results revealed that the isolates of *B. bassiana* did not show significant differences in their ability to cause larval mortality for FAW when applied at a concentration of 1 × 10^6^ conidia/mL and measured at 7 days post-treatment; except the QB-3.45 isolate, which caused ≤10.0% mortality against the second instar larvae of FAW (F_2.89_ = 12; *p* > 0.011). The QB-3.428, QB-3.46 and QB-3.436 isolates of *B. bassiana* caused 13.4% to 14.9% mortality rates of larvae of FAW that were treated with 1 × 10^7^ conidia/mL (F_6.35_ = 12; *p* > 0.000). The QB-3.45 and QB-3.45 isolates of *B. bassiana* caused larval mortality of 25.6 and 20.0% and the QB-3.428 and QB-3.436 isolates caused 17.8 and 14.4% larval mortality of FAW when applied at a concentration of 1 × 10^8^ conidia/mL (F_7.13_ = 12; *p* > 0.000) (Figure 3 and Supplementary Table S5).

### 3.5. Effect of Beauveria bassiana Isolates against Feeding Efficacy of Second Instar FAW Larvae

**Figure 4:**
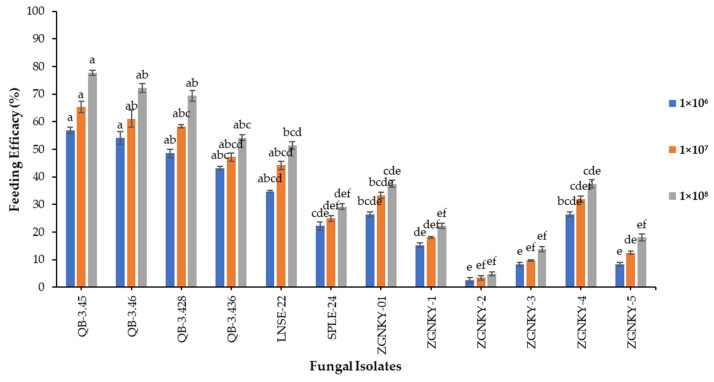
The feeding efficacy of second instar FAW larvae infected with different concentrations of *B. bassiana* isolates. Means followed by the same letters of alphabets are not significantly different according to Tukey’s test at *p* < 0.05.

The results indicated that the QB-3.45 and QB-3.46 isolates of *B. bassiana* significantly reduced the feeding efficacies of the second instar larvae of FAW by 56.9 and 54.2%, respectively, followed by QB-3.428 and QB-3.436, which reduced the feeding efficacy to 48.6 and 43.1% when applied at a concentration of 1 × 10^6^ conidia/mL and measured at 48 h post-treatment (F_17.2_ = 12; *p* > 0.000). The QB-3.45 and QB-3.46 isolates of *B. bassiana* reduced the feeding efficacy to 65.3 and 61.1%, respectively, followed by QB-3.428 (58.3%), in second instar larvae when applied at a concentration of 1 × 10^7^ conidia/mL (F_13.7_ = 12; *p* > 0.000). The QB-3.45 and QB-3.46 isolates of *B. bassiana* reduced the feeding efficacy rates to 77.8 and 72.2%, respectively, followed by QB-3.428 (69.4%), against the second instar FAW larvae (F_24.8_ = 12; *p* > 0.000). The QB-3.436 and LNSE-22 isolates of *B. bassiana* moderately reduced the feeding efficacy to 54.2 and 51.4%, respectively, against the second instar larva of FAW (Figure 4 and Supplementary Table S6).

### 3.6. Effect of B. bassiana Isolates against FAW Pupae

**Figure 5:**
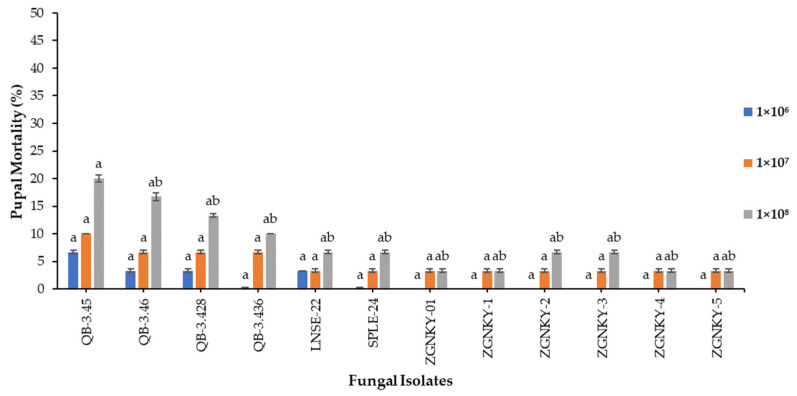
The mortality of pupae infected with different concentrations of *B. bassiana* isolates. Means followed by the same letters are not significantly different according to Tukey’s test at *p* < 0.05.

The results showed that the isolates of *B. bassiana* were not significantly responsible for the causation of the mortality of the pupae of FAW that were treated with 1 × 10^6^ and 1 × 10^7^ conidia/mL conidia/mL. The QB-3.45 and QB-3.46 isolates of *B. bassiana* were found to be effective by causing pupal mortality rates of 20.0 and 16.7% when applied at a concentration of 1 × 10^8^ conidia/mL and measured at 15 days post-treatment (F_2.38_ = 12; *p* > 0.000) (Figure 5 and Supplementary Table S7).

Factorial analysis of variance revealed a significant effect of the conidial concentrations (F_2, 286_ = 280.34; *p* > 0.000), the treatments (F_11, 286_ = 106.20; *p* > 0.000), the life stages (F_3_, 286 = 432.47; *p* > 0.000), and the interaction (F_66, 286_ = 1.66; *p* > 0.002) on the mortality of FAW (Table 2 and Supplementary Table S8).

## 4. Discussion

The present study evaluated the pathogenicity of 12 isolates of *Beauveria bassiana* against invasive FAW. The eggs are immobile and, therefore, were most easily controlled during the egg development process before hatching [75,76,77]. Excessive nutrients are required for the egg development until hatching. In the present study, the isolates of *B. bassiana* caused 87.3% egg mortality in FAW. Our results are supported by those of previous studies, wherein different isolates of *B. bassiana* were found to be able to induce the egg mortality of *S. frugiperda* [61,62,78], *Spodoptera exigua* [79], *Maruca vitrata* [80], *Perileucoptera coffeella* [81] and *Phthorimaea operculella* [82].

Our findings are consistent with those of previous studies wherein different isolates of *B. bassiana* induced FAW neonatal mortality [61,62]. Other isolates of *B. bassiana* significantly induced cumulative mortality in the eggs and neonates of FAW in the present study. The findings of our results are inconsistent with those of previous studies wherein different isolates of *B. bassiana* caused cumulative mortality to the eggs and neonates of FAW [61,62].

Although some fungal isolates were found to be less pathogenic, some of the studied fungal isolates showed effective pathogenicity. The results of a previous study are not consistent with our finding that the isolates of *B. bassiana* caused larval mortality [59]. The pathogenicity of some fungal isolates is directly associated with the stage of the instar, e.g., the fungal isolates proved to be more effective against early instar larvae than later instar lepidopterans larvae [83]. In one study, an isolate of *B. bassiana* caused larval mortality against a second instar of FAW [62]. However, *B. bassiana* that was isolated from soil caused higher larval mortality than the same strain that was isolated from maize [84]. Variations in the effectiveness of *B. bassiana* have also been reported against different instar stages of FAW larvae [85]. In contrast, it was observed that isolates of *B. bassiana* proved to be the least effective against larvae of FAW compared with the other lepidopteran pests [86]. Previous studies reported that the virulence of *B. bassiana* depends on the passage and climate conditions of the strain through certain hosts [87,88,89]. Therefore, more research is needed to explore the mechanisms behind the least pathogenicity of *B. bassiana* isolates against the larvae of FAW in the present study.

Interestingly, one isolate of *B. bassiana* caused significant larval mortality against FAW [44,60]. Another study reported that the presently studied isolates of *B. bassiana* significantly affected the larval growth of *Dendrolimus pini* [90]. The resistance of mature larvae compared with that of young larvae may be due to the composition of the larval integument [91]. Molting may be another factor that is responsible for the lack of inoculum and hence the lower chances of fungal infection [92]. Another challenge the we repeatedly faced was the process of surface-sterilizing the neonates’ (very small) cadavers. If the dead larvae do not show mycosis, then it is difficult to say that they had died due to EPF, because mycosis is the only strong evidence of larval mortality due to EPF. We assumed from the results of the present study that the larvae might have died due to diminished feeding efficacy leading to the physiology of the larvae to be disturbed, which ultimately lead to larval mortality (antifeedant effects).

A variety of toxins, such as beauvericin, bassianin, bassianolide and oxalic acid are secondary metabolites that are produced by *B. bassiana*. *B. bassiana* parasitizes and kills its hosts with the help of these toxins [93]. In the present study, the isolates of *B. bassiana* reduced the feeding efficacy of the FAW larvae [61], this reduction in the feeding efficacy of the larvae that were treated with isolates of *B. bassiana* might be due to the production of these toxins which first parasitize the larvae and then affect the feeding efficacy of larvae, the same as occurs in other insect species [94,95,96,97]. The reduction in the feeding efficacy of the larvae that were treated with the isolates of *B. bassiana* in the present study might be due to the production of these toxins which parasitize the larvae and then the larvae cannot feed on the provided maize leaves efficiently. Our results contrast those of a previous study wherein the feeding efficacy of *Heliothis zea* was not reduced after treatment with isolates of *B. bassiana* [98]. The results of that previous study are consistent with our findings revealing the induced change in the feeding behavior of infected larvae of *D. pini* that were treated with isolates of *B. bassiana* [99]. The reduction in the feeding efficacy of the larvae that are treated with fungi is one of the main reasons for underlying pest mortality, providing evidence for the pathogenicity of fungi, but this phenomenon needs to be further assessed in order to determine the antifeedant effects that can be caused by fungi [100]. Toxins in fungi might cause the mechanical disruption of insects’ structural integrity that leads to the considerable reduction in their feeding efficacy [101].

The isolates of *B. bassiana* were significantly ineffective in inducing the pupal mortality of FAW. It was observed that the early immature stages are more sensitive to the fungal isolates than the later stages of the insects’ growth. Previous studies have supported our findings, wherein isolates of *B. bassiana* did not show significant pathogenicity against the pupae of *Spodoptera litura* [102,103]. Therefore, we recommend implementing control practices at early stages of the larval cycle in order to minimize the damage to maize by invasive FAW.

## 5. Conclusions

The *bassiana* isolates were effective against the immature stages (egg and neonatal larvae) of FAW. These isolates also showed effectiveness in reducing the feeding efficacy of the treated larvae. In the present study, the QB-3.45, QB-3.46 and QB-3.428 isolates of *B. bassiana* proved to be the most effective for the management of the FAW population. Therefore, these isolates perhaps provide bases for the development of commercial biological insecticides. Further research is needed to find the main toxins that affect the virulence factors of the entomopathogenic fungal isolates.

## Figures and Tables

**Table 1:** Information about the isolates of *B. bassiana* tested against FAW in this study.

Isolates	Host	Site of Origin (Country)	Year of Isolation
QB-3.45	*Dendrolimus punctatus punctatus* (Lepidoptera: Dendrolimus)	Guangdong (China)	2014
QB-3.46	*Ostrinia nubilalis* (Lepidoptera: Pyralidae)	Guan, Hebei (China)	2014
QB-3.428	*Ostrinia nubilalis* (Lepidoptera: Pyralidae)	Changchun, Jilin (China)	2014
QB-3.436	*Nilaparvata lugens* (Homoptera: Delphacidae)	Philippines	2013
LNSE-22	*Ostrinia nubilalis* (Lepidoptera: Pyralidae)	Guangdong (China)	2019
SPLE-24	*Ostrinia nubilalis* (Lepidoptera: Pyralidae)	Guangdong (China)	2019
ZGNKY-01	*Ostrinia nubilalis* (Lepidoptera: Pyralidae)	Urumqi, Xinjiang (China)	2015
ZGNKY-1	*Ostrinia nubilalis* (Lepidoptera: Pyralidae)	Beijing (China)	2015
ZGNKY-2	*Ostrinia nubilalis* (Lepidoptera: Pyralidae)	Beijing (China)	2015
ZGNKY-3	*Ostrinia nubilalis* (Lepidoptera: Pyralidae)	Beijing (China)	2015
ZGNKY-4	*Ostrinia nubilalis* (Lepidoptera: Pyralidae)	Suining, Liaoning (China)	2015
ZGNKY-5	*Ostrinia nubilalis* (Lepidoptera: Pyralidae)	Chaozhou, Guangdong (China)	2015

**Table 2:** Percent mortality (Means ± SE) of immature stages of FAW treated with different concentrations of isolates of *Beauveria bassiana*.

	Percent Mortality ± Means Standard Error				
Concentrations	Isolates	Eggs ^A^	Neonates ^B^	Larvae ^C^	Pupae ^D^
1 × 10^6^ Conidia/mL ^C^	QB-3.45 ^A^	30.0 ± 1.7 a	9.5 ± 0.3 ab	10.0 ± 1.0 a	6.7 ± 0.3 a
	QB-3.46 ^AB^	25.3 ± 1.2 a	13.4 ± 2.1 ab	8.9 ± 1.2 a	3.3 ± 0.3 a
	QB-3.428 ^B^	28.7 ± 1.8 a	14.9 ± 1.5 a	7.8 ± 0.9 a	3.3 ± 0.3 a
	QB-3.436 ^C^	20.7 ± 1.5 ab	13.4 ± 1.2 ab	7.8 ± 0.7 a	3.3 ± 0.3 a
	LNSE-22 ^D^	24.0 ± 1.5 a	9.6 ± 0.9 ab	4.4 ± 0.9 a	0.0 ± 0.0 a
	SPLE-24 ^E^	10.7 ± 0.9 bc	6.7 ± 1.2 ab	4.4 ± 0.3 a	3.3 ± 0.3 a
	ZGNKY-01 ^E^	11.3 ± 0.7 bc	4.5 ± 1.0 ab	1.1 ± 0.3 a	0.0 ± 0.0 a
	ZGNKY-1 ^FG^	4.0 ± 0.6 c	6.3 ± 0.6 ab	1.1 ± 0.3 a	0.0 ± 0.0 a
	ZGNKY-2 ^G^	5.3 ± 1.5 c	3.5 ± 0.3 ab	1.1 ± 0.3 a	0.0 ± 0.0 a
	ZGNKY-3 ^G^	2.0 ± 0.6 c	4.8 ± 0.9 ab	1.1 ± 0.3 a	0.0 ± 0.0 a
	ZGNKY-4 ^EF^	8.0 ± 1.5 c	2.2 ± 0.6 ab	2.2 ± 0.3 a	0.0 ± 0.0 a
	ZGNKY-5 ^G^	3.3 ± 0.9 c	1.4 ± 0.7 b	1.1 ± 0.3 a	0.0 ± 0.0 a
1 × 10^7^ Conidia/mL ^B^	QB-3.45 ^A^	87.3 ± 1.5 a	47.6 ± 1.2 ab	9.5 ± 0.3 a	10.0 ± 0.0 a
	QB-3.46 ^AB^	82.7 ± 2.4 a	53.6 ± 1.5 a	13.4 ± 2.1 ab	6.7 ± 0.3 a
	QB-3.428 ^B^	79.3 ± 2.2 a	45.3 ± 0.9 a	14.9 ± 1.5 abc	6.7 ± 0.3 a
	QB-3.436 ^C^	56.0 ± 2.3 b	34.8 ± 1.2 abc	13.4 ± 1.2 abc	6.7 ± 0.3 a
	LNSE-22 ^D^	50.0 ± 2.0 bc	30.7 ± 1.2 abcd	9.6 ± 0.9 abc	3.3 ± 0.3 a
	SPLE-24 ^E^	30.7 ± 0.3 def	20.2 ± 1.2 bcde	6.7 ± 1.2 bc	3.3 ± 0.3 a
	ZGNKY-01 ^E^	39.3 ± 2.2 bcd	17.6 ± 0.7 cde	4.5 ± 1.0 bc	3.3 ± 0.3 a
	ZGNKY-1 ^FG^	20.0 ± 2.3 efg	15.8 ± 1.2 cde	6.3 ± 0.6 c	3.3 ± 0.3 a
	ZGNKY-2 ^G^	12.7 ± 1.5 fg	12.2 ± 1.2 cde	3.5 ± 0.3 c	3.3 ± 0.3 a
	ZGNKY-3 ^G^	15.3 ± 1.5 fg	10.2 ± 1.5 de	4.8 ± 0.9 c	3.3 ± 0.3 a
	ZGNKY-4 ^EF^	35.3 ± 1.5 cde	10.3 ± 0.9 cde	2.2 ± 0.6 bc	3.3 ± 0.3 a
	ZGNKY-5 ^G^	18.7 ± 2.0 efg	8.2 ± 0.9 de	1.4 ± 0.7 c	3.3 ± 0.3 a
1 × 10^8^ Conidia/mL ^A^	QB-3.45 ^A^	70.0 ± 1.2 a	20.0 ± 0.6 abcd	25.6 ± 1.2 a	20.0 ± 0.6 a
	QB-3.46 ^AB^	64.7 ± 1.9 ab	34.6 ± 0.9 a	20.0 ± 1.0 ab	16.7 ± 0.7 ab
	QB-3.428 ^B^	54.7 ± 1.8 b	30.8 ± 1.5 a	17.8 ± 0.9 abc	13.3 ± 0.3 ab
	QB-3.436 ^C^	40.0 ± 2.3 c	25.6 ± 1.2 ab	14.4 ± 1.5 abcd	10.0 ± 0.0 ab
	LNSE-22 ^D^	35.3 ± 1.2 c	21.7 ± 0.6 abc	10.0 ± 1.0 bcd	6.7 ± 0.3 ab
	SPLE-24 ^E^	15.3 ± 1.2 de	12.6 ± 0.9 bcde	10.0 ± 1.0 bcd	6.7 ± 0.3 ab
	ZGNKY-01 ^E^	18.7 ± 0.9 d	10.6 ± 0.9 bcde	7.8 ± 0.3 bcd	3.3 ± 0.3 ab
	ZGNKY-1 ^FG^	10.0 ± 1.2 de	9.6 ± 0.9 cde	5.6 ± 0.3 cd	3.3 ± 0.3 ab
	ZGNKY-2 ^G^	6.7 ± 0.9 de	7.9 ± 1.2 cde	4.4 ± 0.3 cd	6.7 ± 0.3 ab
	ZGNKY-3 ^G^	8.0 ± 0.6 de	7.2 ± 0.7 cde	5.6 ± 0.7 cd	6.7 ± 0.3 ab
	ZGNKY-4 ^EF^	17.3 ± 2.3 d	5.6 ± 0.3 cde	12.2 ± 0.3 abcd	3.3 ± 0.3 ab
	ZGNKY-5 ^G^	8.7 ± 0.9 de	3.6 ± 0.9 de	3.3 ± 0.0 d	3.3 ± 0.3 ab

For each concentration, values within a column followed by the same small letters are not significantly different (one-way ANOVA; HSD post-hoc test at *p* ≤ 0.05). Capital letters along conidial concentration isolate or FAW’s immature stages indicate the statistical difference among the treatments (factorial ANOVA; HSD post-hoc test at *p* ≤ 0.05).

## Data Availability

The data that are presented in this study are available in the article.

## Supplementary Materials

The following supporting information can be downloaded at: https://www.mdpi.com/article/10.3390/jof8070717/s1. Table S1: Germination rates of the *B. bassiana* isolates used in this study against FAW; Table S2: Cumulative mortality of eggs and neonate larvae of *S. frugiperda* induced by *B. bassiana* isolates treated with 1 × 10^6^ conidia/mL; Table S3: Cumulative mortality of eggs and neonate larvae of FAW induced by *B. bassiana* isolates treated with 1 × 10^7^ conidia/mL; Table S4: Cumulative mortality of eggs and neonate larvae of FAW induced by *B. bassiana* isolates treated with 1 × 10^8^ conidia/mL; Table S5: Mortality of second instar larvae of FAW infected with different concentrations of *B. bassiana* isolates; Table S6: Feeding efficacy of second instar larvae of FAW infected with different concentrations of *B. bassiana* isolates; Table S7: Mortality of pupae of FAW infected with different concentrations of *B. bassiana* isolates and Table S8: Analysis of variance comparison table for mean percent mortality of eggs, neonate, larvae and pupae of FAW treated with different concentrations of isolates of *B. bassiana* under laboratory conditions.
