# Supplementary material for: Bioassays of Beauveria bassiana Isolates against the Fall Armyworm, Spodoptera frugiperda
Source: J Fungi (Basel). 2022 Jul 8;8(7):717. doi: 10.3390/jof8070717 (PMC9324617; doi:10.3390/jof8070717)
Supplement: Supplementary file 1 [file jof-08-00717-s001.zip › jof-1756944-supplementary.pdf]

Supplementary Table S1. Germination rates of the *B. bassiana* isolates used in this study against FAW.

Isolates	Percent Germination ± Means Standard Error		
	**1 x 10[6] Conidia/mL**	**1 x 10[7] Conidia/mL**	**1 x 10[8] Conidia/mL**
QB-3.45	90.3 ± 0.9	91.3 ± 2.4	90.0 ± 1.5
QB-3.46	90.0 ± 1.2	90.7 ± 1.2	90.0 ± 1.2
QB-3.428	91.7 ± 1.2	92.7 ± 0.9	91.3 ± 0.7
QB-3.436	90.7 ± 0.9	90.7 ± 0.9	90.0 ± 0.6
LNSE-22	90.7 ± 0.9	91.3 ± 2.8	92.7 ± 0.7
SPLE-24	90.0 ± 2.3	90.7 ± 1.5	93.0 ± 1.2
ZGNKY-01	91.0 ± 2.1	91.3 ± 2.7	91.7 ± 1.8
ZGNKY-1	90.3 ± 0.9	91.0 ± 1.5	93.0 ± 1.0
ZGNKY-2	90.3 ± 1.2	91.0 ± 2.1	93.7 ± 1.8
ZGNKY-3	91.7 ± 1.2	90.0 ± 0.6	92.7 ± 1.8
ZGNKY-4	91.7 ± 1.8	90.3 ± 2.3	91.3 ± 1.9
ZGNKY-5	91.0 ± 1.2	90.0 ± 1.5	92.0 ± 1.5

Supplementary Table S2. Cumulative mortality of eggs and neonate larvae of *S. frugiperda* induced by *B. bassiana* isolates treated with 1 × 10[6] conidia/ml.

Isolates	Percent Mortality ± Means Standard Error		
	**Eggs Mortality**	**Neonates Mortality**	**Cumulative Mortality**
QB-3.45	30.0 ± 1.7 a	9.5 ± 0.3 ab	36.7 ± 1.8 a
QB-3.46	25.3 ± 1.2 a	13.4 ± 2.1 ab	35.3 ± 2.8 a
QB-3.428	28.7 ± 1.8 a	14.9 ± 1.5 a	39.3 ± 2.7 a
QB-3.436	20.7 ± 1.5 ab	13.4 ± 1.2 ab	31.3 ± 2.2 ab
LNSE-22	24.0 ± 1.5 a	9.6 ± 0.9 ab	31.3 ± 1.8 ab
SPLE-24	10.7 ± 0.9 bc	6.7 ± 1.2 ab	16.7 ± 0.9 bc
ZGNKY-01	11.3 ± 0.7 bc	4.5 ± 1.0 ab	15.3 ± 1.7 bc
ZGNKY-1	4.0 ± 0.6 c	6.3 ± 0.6 ab	10.0 ± 1.0 c
ZGNKY-2	5.3 ± 1.5 c	3.5 ± 0.3 ab	8.7 ± 1.5 c
ZGNKY-3	2.0 ± 0.6 c	4.8 ± 0.9 ab	6.7 ± 0.3 c
ZGNKY-4	8.0 ± 1.5 c	2.2 ± 0.6 ab	10.0 ± 2.0 c
ZGNKY-5	3.3 ± 0.9 c	1.4 ± 0.7 b	4.7 ± 0.3 c
F	19.5	3.63	16.1
df	12	12	12
P<	0.000	0.002	0.000

Means ± SE within a column not sharing common letters are significantly different using Tuckey's test at $p < 0.05$.

Supplementary Table S3. Cumulative mortality of eggs and neonate larvae of FAW induced by *B. bassiana* isolates treated with 1×10^7 conidia/ml.

Isolates	Percent Mortality ± Means Standard Error		
	**Eggs Mortality**	**Neonates Mortality**	**Cumulative Mortality**
QB-3.45	87.3 ± 1.5 a	47.6 ± 1.2 ab	93.3 ± 0.3 a
QB-3.46	82.7 ± 2.4 a	53.6 ± 1.5 a	92.0 ± 1.5 a
QB-3.428	79.3 ± 2.2 a	45.3 ± 0.9 a	88.7 ± 1.5 a
QB-3.436	56.0 ± 2.3 b	34.8 ± 1.2 abc	71.3 ± 1.2 b
LNSE-22	50.0 ± 2.0 bc	30.7 ± 1.2 abcd	65.3 ± 0.9 bc
SPLE-24	30.7 ± 0.3 def	20.2 ± 1.2 bcde	44.7 ± 1.2 de
ZGNKY-01	39.3 ± 2.2 bcd	17.6 ± 0.7 cde	50.0 ± 2.5 cd
ZGNKY-1	20.0 ± 2.3 efg	15.8 ± 1.2 cde	32.7 ± 1.7 ef
ZGNKY-2	12.7 ± 1.5 fg	12.2 ± 1.2 cde	23.3 ± 2.2 f
ZGNKY-3	15.3 ± 1.5 fg	10.2 ± 1.5 de	24.0 ± 1.2 f
ZGNKY-4	35.3 ± 1.5 cde	10.3 ± 0.9 cde	38.7 ± 1.9 def
ZGNKY-5	18.7 ± 2.0 efg	8.2 ± 0.9 de	25.3 ± 1.9 f
F	60.3	11.6	90.3
df	12	12	12
$P<$	0.000	0.000	0.000

Means ± SE within a column not sharing common letters are significantly different using Tuckey's test at $p < 0.05$.

Supplementary Table S4. Cumulative mortality of eggs and neonate larvae of FAW induced by *B. bassiana* isolates treated with 1×10^8 conidia/ml.

Isolates	Percent Mortality ± Means Standard Error		
	**Eggs Mortality**	**Neonates Mortality**	**Cumulative Mortality**
QB-3.45	70.0 ± 1.2 a	20.0 ± 0.6 abcd	76.0 ± 1.5 a
QB-3.46	64.7 ± 1.9 ab	34.6 ± 0.9 a	77.3 ± 2.0 a
QB-3.428	54.7 ± 1.8 b	30.8 ± 1.5 a	68.7 ± 2.3 ab
QB-3.436	40.0 ± 2.3 c	25.6 ± 1.2 ab	55.3 ± 1.7 bc
LNSE-22	35.3 ± 1.2 c	21.7 ± 0.6 abc	49.3 ± 0.9 c
SPLE-24	15.3 ± 1.2 de	12.6 ± 0.9 bcde	26.0 ± 2.1 d
ZGNKY-01	18.7 ± 0.9 d	10.6 ± 0.9 bcde	27.3 ± 1.8 d
ZGNKY-1	10.0 ± 1.2 de	9.6 ± 0.9 cde	18.7 ± 2.0 de
ZGNKY-2	6.7 ± 0.9 de	7.9 ± 1.2 cde	14.0 ± 2.1 de
ZGNKY-3	8.0 ± 0.6 de	7.2 ± 0.7 cde	14.7 ± 1.2 de
ZGNKY-4	17.3 ± 2.3 d	5.6 ± 0.3 cde	22.0 ± 2.6 d
ZGNKY-5	8.7 ± 0.9 de	3.6 ± 0.9 de	12.0 ± 1.5 de
F	69.9	10.3	52.5
df	12	12	12
P<	0.000	0.000	0.000

Means ± SE within a column not sharing common letters are significantly different using Tuckey's test at $p < 0.05$.

Supplementary Table S5. Mortality of second instar larvae of FAW infected with different concentrations of *B. bassiana* isolates.

Beauveria bassiana **Isolates**	**Percent Mortality ± Means Standard Error**		
	**1 x 10^6 conidia/mL**	**1 x 10^7 conidia/mL**	**1 x 10^8 conidia/mL**
QB-3.45	10.0 ± 1.0 a	9.5 ± 0.3 a	25.6 ± 1.2 a
QB-3.46	8.9 ± 1.2 a	13.4 ± 2.1 ab	20.0 ± 1.0 ab
QB-3.428	7.8 ± 0.9 a	14.9 ± 1.5 abc	17.8 ± 0.9 abc
QB-3.436	7.8 ± 0.7a	13.4 ± 1.2 abc	14.4 ± 1.5 abcd
LNSE-22	4.4 ± 0.9 a	9.6 ± 0.9 abc	10.0 ± 1.0 bcd
SPLE-24	4.4 ± 0.3 a	6.7 ± 1.2 bc	10.0 ± 1.0 bcd
ZGNKY-01	1.1 ± 0.3 a	4.5 ± 1.0 bc	7.8 ± 0.3 bcd
ZGNKY-1	1.1 ± 0.3 a	6.3 ± 0.6 c	5.6 ± 0.3 cd
ZGNKY-2	1.1 ± 0.3 a	3.5 ± 0.3 c	4.4 ± 0.3 cd
ZGNKY-3	1.1 ± 0.3 a	4.8 ± 0.9 c	5.6 ± 0.7 cd
ZGNKY-4	2.2 ± 0.3 a	2.2 ± 0.6 bc	12.2 ± 0.3 abcd
ZGNKY-5	1.1 ± 0.3 a	1.4 ± 0.7 c	3.3 ± 0.0 d
F	2.89	6.35	7.13
df	12	12	12
P<	0.011	0.000	0.000

Means ± SE within a column not sharing common letters are significantly different using Tuckey's test at $p < 0.05$.

Supplementary Table S6. Feeding efficacy of second instar larvae of FAW infected with different concentrations of *B. bassiana* isolates.

Beauveria bassiana **Isolates**	**Percent Feeding Efficacy ± Means Standard Error**		
	**1 x 10^6 conidia/mL**	**1 x 10^7 conidia/mL**	**1 x 10^8 conidia/mL**
QB-3.45	56.9 ± 1.2 a	65.3 ± 2.0 a	77.8 ± 0.9 a
QB-3.46	54.2 ± 2.3 a	61.1 ± 3.2 ab	72.2 ± 1.5 ab
QB-3.428	48.6 ± 1.5 ab	58.3 ± 0.6 abc	69.4 ± 1.9 ab
QB-3.436	43.1 ± 0.7 abc	47.2 ± 1.5 abcd	54.2 ± 1.0 abc
LNSE-22	34.7 ± 0.3 abcd	44.2 ± 1.5 abcd	51.4 ± 1.3 bcd
SPLE-24	22.2 ± 1.5 cde	25.0 ± 1.0 def	29.2 ± 1.0 def
ZGNKY-01	26.4 ± 0.9 bcde	33.3 ± 1.2 bcde	37.5 ± 1.2 cde
ZGNKY-1	15.3 ± 0.7 de	18.1 ± 0.3 def	22.2 ± 0.9 ef
ZGNKY-2	2.6 ± 0.9 e	3.4 ± 0.9 ef	4.8 ± 0.7 ef
ZGNKY-3	8.3 ± 0.6 e	9.7 ± 0.3 ef	13.9 ± 0.9 ef
ZGNKY-4	26.4 ± 0.9 bcde	31.9 ± 1.2 cdef	37.5 ± 1.5 cde
ZGNKY-5	8.3 ± 0.6 e	12.5 ± 0.6 ef	18.1 ± 1.2 ef
F	17.2	13.7	24.8
df	12	12	12
P<	0.000	0.000	0.000

Means ± SE within a column not sharing common letters are significantly different using Tuckey's test at $p < 0.05$.

Supplementary Table S7. Mortality of pupae of FAW infected with different concentrations of *B. bassiana* isolates

Beauveria bassiana **Isolates**	**Percent Mortality ± Means Standard Error**		
	**1 x 10^6 conidia/mL**	**1 x 10^7 conidia/mL**	**1 x 10^8 conidia/mL**
QB-3.45	6.7 ± 0.3 a	10.0 ± 0.0 a	20.0 ± 0.6 a
QB-3.46	3.3 ± 0.3 a	6.7 ± 0.3 a	16.7 ± 0.7 ab
QB-3.428	3.3 ± 0.3 a	6.7 ± 0.3 a	13.3 ± 0.3 ab
QB-3.436	3.3 ± 0.3 a	6.7 ± 0.3 a	10.0 ± 0.0 ab
LNSE-22	0.0 ± 0.0 a	3.3 ± 0.3 a	6.7 ± 0.3 ab
SPLE-24	3.3 ± 0.3 a	3.3 ± 0.3 a	6.7 ± 0.3 ab
ZGNKY-01	0.0 ± 0.0 a	3.3 ± 0.3 a	3.3 ± 0.3 ab
ZGNKY-1	0.0 ± 0.0 a	3.3 ± 0.3 a	3.3 ± 0.3 ab
ZGNKY-2	0.0 ± 0.0 a	3.3 ± 0.3 a	6.7 ± 0.3 ab
ZGNKY-3	0.0 ± 0.0 a	3.3 ± 0.3 a	6.7 ± 0.3 ab
ZGNKY-4	0.0 ± 0.0 a	3.3 ± 0.3 a	3.3 ± 0.3 ab
ZGNKY-5	0.0 ± 0.0 a	3.3 ± 0.3 a	3.3 ± 0.3 ab
F	1.13	0.67	2.38
df	12	12	12
P<	0.377	0.766	0.031

Means ± SE within a column not sharing common letters are significantly different using Tuckey's test at $p < 0.05$.

Supplementary Table S8. Analysis of variance comparison table for mean percent mortality of eggs, neonate, larvae and pupae of FAW treated with different concentrations of isolates of *B. bassiana* under laboratory conditions.

Source	DF	SS	MS	F	P
Concentrations	2	16776	8387.8	280.34	0.0000
Treatments	11	34736	3157.8	106.20	0.0000
Life Stages	3	38577	12859.1	432.47	0.0000
Conc*Treat	22	4979	226.3	7.56	0.0000
Conc*Stages	6	6175	1029.1	34.40	0.0000
Treat*Stages	33	19012	576.1	19.26	0.0000
Conc*Treat*Stages	66	3285	49.8	1.66	0.0025
Error	286	8557	29.9		
Total	431	132103			
Grand Mean	14.451				
CV	37.85				
